# Normal pace walking is beneficial to young participants’ executive abilities

**DOI:** 10.1186/s13102-022-00587-y

**Published:** 2022-11-19

**Authors:** J. N. Zhang, L. S. Xiang, Y. Shi, F. Xie, Y. Wang, Y. Zhang

**Affiliations:** Department of Rehabilitation Medicine, Third Affiliated Hospital of Soochow University, Changzhou, 213003 Jiangsu Province China

**Keywords:** Walking, Stroop test, Executive function, Near-infrared spectroscopy (NIRS), Prefrontal cortex (PFC)

## Abstract

**Background:**

Exercise can improve cognitive function. The impact of acute exercise on cognition is related to exercise intensity. This study aimed to explore whether normal walking had a beneficial effect on cognition.

**Methods:**

Compared with standing still, thirty healthy young men walked on a treadmill at a normal pace, and completed the Stroop test. Near-infrared spectroscopy was used to monitor the hemodynamic changes of the prefrontal cortex during the entire experiment.

**Results:**

Studies showed that normal walking did not stimulate higher average cerebral oxygen in the PFC, but the peak cerebral oxygen in cognitive tests during walking was higher (Stroop Word: 2.56 ± 0.43 and 3.80 ± 0.50, *P* < 0.01, Stroop Color: 2.50 ± 0.37 and 3.66 ± 0.59, *P* < 0.05, Stroop Color-Word: 4.13 ± 0.55 and 5.25 ± 0.66, *P* < 0.01, respectively), and better results were achieved in the Stroop Color-Word test, which was reflected in faster reaction times (49.18 ± 1.68 s, 56.92 ± 2.29 s, respectively, *P* < 0.001) and higher accuracies (46.19 ± 0.69, 44.15 ± 0.91, respectively, *P* = 0.018).

**Conclusion:**

For healthy young people, even a normal walk is therefore good for cognition.

## Background

Exercise has a beneficial effect on cognitive functions [1, 2]. Different exercise intensities and durations, and types of cognitive tasks have differing degrees of influence [1, 3]. Chronic long-term exercise and acute physical exercise are beneficial to the improvement of cognitive function. Long-term aerobic exercise improves cognitive function, including executive abilities, by improving cerebrovascular regulation [4]. A single bout of acute physical exercise also directly affects the cerebral blood flow [5], and has beneficial effects on cognitive abilities [6].

The intensities of a single acute exercise affect cognition. According to the American College of Sports Medicine (ACSM) guidelines [7], to improve cardiorespiratory endurance and the intensity of muscle aerobic metabolism, the target heart rate of medium-to-high-intensity aerobic exercise should reach 40% or more of the reserve heart rate. At this time, the output of the heart per minute is higher than in the resting state, and the cerebral blood flow is increased, thereby increasing the activation and effective use of the task-related areas of the prefrontal cortex, to improve cognitive functions [3]. Due to the inverted U-shaped relationships between brain oxygenation and exercise intensity, as exercise intensity further increases, blood flow to the brain and oxygen delivery may also decrease as cardiac output decreases [8]. Compared with moderate intensity exercise, high intensity exercise may therefore actually impair cognitive function due to a decrease in cerebral oxygenation [9].

Compared to running, walking is a common and relaxing low intensity mode of aerobic exercise. Respiratory rhythm and body temperature do not increase during exercise, and the heart rate reserve is ≤ 30%, which has little effect on cardiac output. Walking is a common exercise mode for many elderly people or patients with chronic heart and lung diseases. Pang et al. reported that long-term functional community walking (8 weeks) was beneficial to the cognitive function of elderly patients with stroke [10]. However, it is not known if low intensity exercise like walking has the same acute beneficial effects on cognitive function as medium to high intensity exercise.

Near-infrared spectroscopy (NIRS) acquaints and analyzes such phenomena in real time. Increased regional brain activity is associated with greater local blood flow, and NIRS provides a method for measuring neural activity associated with local changes in hemoglobin (Hb) concentration [11]. By exploiting the different optical absorption curves of oxyhemoglobin (oxyHb) and deoxyhemoglobin (deoxyHb), NIRS can monitor tissue metabolism and brain activity [12] with accuracy comparable to magnetic resonance imaging (MRI) [13]. However, unlike fMRI, NIRS is portable, non-invasive, places fewer behavioral constraints on subjects, and is much less costly [14].

Because the ability to walk and perform cognitive tasks at the same time is a key aspect of daily life, this study designed a dual-task (DT) experiment involving a walking task and a cognitive task, and assuming that executive function during DT was better [15]. To test this hypothesis, subjects' cognitive assessment scores were recorded during single-task (ST) and DT. Cognitive (Stroop test) and motor tasks (normal walking) were performed as ST and simultaneously as DT, respectively. Since the prefrontal cortex (PFC) plays an important role in motor control, cognition and dual task performance [16, 17], NIRS was used to monitor the hemodynamic changes of the PFC in real time to compare cerebral oxygen parameters of the PFC under different task conditions and analyze the influence of walking on executive ability.

## Methods

### Subjects

Thirty right-handed participants aged 19–33 years (14 males and 16 females), with an average formal education of 15 ± 0.48 years, were recruited. The participants had sufficient sleep (> 7 h), and did not drink alcohol or take drugs on the day of testing. All participants had normal or corrected vision, had no color discrimination disorders, and no known history of bone and joint disease or cardiopulmonary disease. Each participant provided informed written consent, and the research protocol was approved by the Ethics Committee of the Third Affiliated Hospital of Soochow University (approval number: 2020–146), and comply with the Declaration of Helsinki. All methods are complied with the Declaration of Helsinki.

### Stroop Tset

The Stroop test is a color naming test developed by Stroop (1953). Subjects are asked to identify and state the ink color as soon as possible based on specific conditions. The test consists of three parts: the Stroop Word, Stroop Color, and Stroop Color-Word tests.

Color names (red, blue, and green) in the Stroop Word experiment were written in black ink, which was the reading task.

Stroop color: the color name was in the circle of the same color, which was a named task.

Stroop Color-Word: the color name was printed with different colored ink (e.g., “blue” printed using green ink), which was the inhibition condition task.

Each section included 50 experiments, which were displayed on paper. The participants were asked to name the experiment from left to right (10 columns) and top to bottom (5 rows). The test order of the 3 parts was random. The total reaction times and correct numbers (modifications do not count) were recorded by two professionally trained investigators. If the scores were inconsistent, a third person was also consulted.

### Hemodynamic data collection

We used a dual-channel NIRS system (EGOS-600A; Enginmed Bio-Medical Electronics, Suzhou, China), equipped with built-in LEDs with near-infrared wavelengths of 760, 810, and 840 nm. According to the Beer-Lambert law, Δ[oxy-Hb] and Δ[deoxy-Hb] were calculated, to measure the hemodynamic responses of the participants’ foreheads. In these experiments, Δ[oxy-Hb] values were more reliable and sensitive to exercise-related changes in cerebral blood flow [18], which were used to characterize changes in the PFC. At the same time, the tissue hemoglobin index (THI) was monitored, and its value change reflected the change rate of local total hemoglobin. Both blood volume and hematocrit ratio can have a positive effect on it, reflecting whether the local tissue is ischemia [19].

**Fig. 1:**
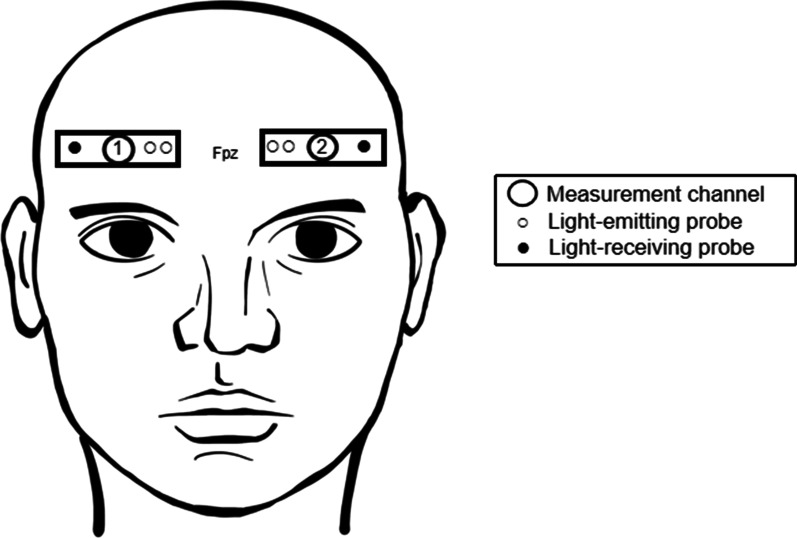
2 measurement channels

According to the international 10–20 electrode system, two pairs of light detectors were placed at Fp1-F7 and Fp2-F8 (Fig. 1), corresponding to light sources placed at Fp1 and Fp2 at a distance of 3 cm and 4 cm from the two detectors, respectively. To detect hemodynamic parameters in tissues with a depth of 2–3 cm, the investigators helped the individuals wear and fix the NIRS probe for each participant. Before wearing, the forehead was wiped with an alcohol-soaked cotton ball, and the participant’s hair was arranged to reduce the influences of oil and cosmetics. The outside of the probe was fixed by a bandage to reduce interference from natural light and to prevent the probe from falling during the experiment. The equipment used multi-channel π filter and high precision AD converter to reduce noise interferences. The probe was worn during the entire experiment, with sampling of the device every 2 s.

### Gait parameter acquisition

To provide a quiet- and interference-free test environment and to reduce the impact on cognitive testing and cerebral oxygen parameters, we chose to complete the test indoors. Due to a limitation of test space, to reduce the influence of turning while walking, we chose to use a treadmill. Confirmation of the running speed of the treadmill was conducted during the preparation phase. The participants were asked to walk on the treadmill at the same speed as their daily walks, using a finger pulse oximeter (YX306 Finger Clip Pulse Oximeter; YuWell, Danyang, China) to monitor their heart rate below 57% HRMax [207 − (0.67 × age)] [20], RPE < 9, to determine the treadmill speed according to the participant’s subjective feelings and heart rates.

We used the Gait Analysis System (OptoGait, Microgate, Microgate S.r.l Via Waltraud Gebert Deeg 3/E IT-39100 Bolzano Italy), with cameras and infrared sensing devices around the treadmill record the subject's walking process in real time, and the program automatically analyzes gait parameters, which collects included the step length of the left and right feet, stride length, gait speed etc. (Step length: Average distance from the ground on one foot to the ground on the opposite foot; Stride length: The distance from one foot landing to the same foot landing again; Step time: Average time from one foot to the other; Stride time: The sum of the time of the support phase and the swing phase.)

### Rating of perceived exertion

Perceived effort rating (RPE) [21] is a subjective exercise effort rating developed by Borg. We used the Borg scale to evaluate the exertion of the participant, ranging from 6 to 20. A score of 7–11 represented “very, very light to very light effort”, and a score of 13–14 represented “a little effort.” A score of 15–19 represented “very, very hard work”, and 20 represented “work harder to the greatest extent.”

### Procedures

The participants had 15 min to adapt before the start of the experiment, involving familiarity with walking on the treadmill, adjustment of the appropriate speed, understanding the Stroop test rules, and completing the exercise (practice version).

### ST

*Cognitive tasks*: Subjects were asked to complete the Stroop test while standing still. The Stroop test consisted of 50 words or color blocks with a size of 4 * 4 cm (10 columns, 5 rows), with a spacing of 1.5 cm between each column and 3 cm between each row. The test cardboard was placed 0.5–1 m in front of the subject. The subject's visual acuity (or corrected visual acuity) had been confirmed before the experiment to complete the test.

*Motor tasks*: According to the walking speed tested by each subject before the experiment, the subjects walked on the treadmill at a constant speed. The walking recording time was 5 min.

### DT

Subjects were required to complete the Stroop test while walking at a constant pace on a treadmill. The size and arrangement of the test boards were the same as those of cognitive ST. The Stroop test version used was different, and the A or B version is randomly selected in the ST and DT stages. Walking time depended on how long the subject completed the Stroop test.

The following steps were then completed, during which the PFC oxygenation was measured. (1) Measuring the baseline values of brain oxygen parameters in the standing position for 5 min, (2) measuring the cognitive ST, (3) measuring the walking ST, and (4) performing both cognitive and walking tasks at the same time, using the dual-task evaluation RPE from beginning to end. The step order of (2)–(4) was random, and there was a 5-min rest period after each task.

The experiments were conducted in a quiet and isolated room. Air conditioning was adjusted to maintain a temperature 20–24 °C to reduce the influence of sweating on the sensitivity of the instrument probe.

### Statistical analysis

The participant’s average Δ[oxy-Hb] and Δ[deoxy-Hb] values during the baseline level were counted, with the Stroop test alone, walking, and the Stroop test during walking, to compare whether there were differences in cerebral hemodynamics at different task stages. The first 10% of each piece of the Hemodynamic data collected by NIRS were removed to reduce interference of the previous state. One-way analysis of variance was used to analyze differences in brain oxygen parameters between groups at each task stage, and the data were tested for homogeneity of variance (*P* > 0.05), using analysis of variance and Bonferroni analysis for multiple comparisons. The paired t-test was used to analyze the left and right side PFC brain oxygen data, Stroop times, correct numbers, and the walking parameter comparisons between ST and DT. The Shapiro–Wilk’s test was used to test the normality of the parameter distribution.

All statistical analyses used SPSS statistical software for Windows, version 26.0 (SPSS, Chicago, IL, USA) and figures were constructed using Prism 8.0 software (GraphPad, San Diego, CA, USA). Statistical significance was designed as *P* < 0.05.

## Results

### Behavioral data

All 30 participants completed the experiments with a walking speed of 2.26 ± 0.41 km/h. The gait parameters of walking ST and DT are shown in Table 1. The results showed that gait parameters (step length, stride length, step time, etc.) were not significantly different between walking ST and DT.

**Table 1:** Gait spatiotemporal parameters for walking ST and DT group

Spatiotemporal parameters	Walking ST group		DT group		*P*
	Mean	SD	Mean	SD	
Step length (metres)					
Left	0.766	0.146	0.762	0.131	0.684
Right	0.767	0.147	0.763	0.131	0.642
Stride length (metres)	1.534	0.292	1.525	0.262	0.641
Step time (seconds)					
Left	1.422	0.310	1.420	0.278	0.940
Right	1.433	0.323	1.418	0.275	0.474
Stride time (seconds)	2.873	0.645	2.845	0.598	0.440

### Stroop test data

**Fig. 2:**
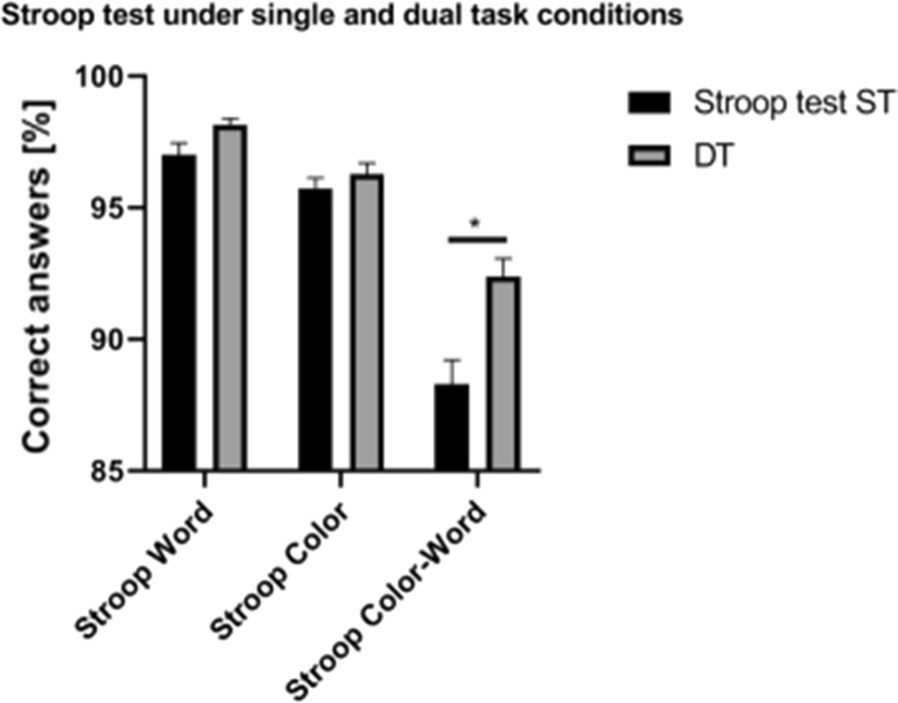
Stroop test under single and dual task conditions. *P < 0.05

Dual-task cognitive performance of Stroop Color-Word was better than single-task performance (Fig. 2). When considering the advanced degree of education of the participants, to avoid the ceiling effect, when counting the correct number, cases that were all correct were deleted.

Using the Stroop word test, the DT time (21.14 ± 0.72 s) was shorter than that of the ST time (21.28 ± 0.98 s), and the correct number of DTs (49.08 ± 0.23) was greater than that of the ST (48.50 ± 0.45) (removal of all correctness, and a total of 12 cases were analyzed), but the difference was not statistically significant (*P* = 0.640 and 0.294, respectively).

Using the Stroop color test, the DT time (31.96 ± 1.32 s) was shorter than that of the ST time (32.37 ± 1.22 s), and the correct number of DTs (48.14 ± 0.41) was greater than that of the ST (47.86 ± 0.41) (22 cases analyzed), but the difference was not statistically significant (*P* = 0.594 and 0.650, respectively).

Using the Stroop Color-Word test, the DT time (49.18 ± 1.68 s) was shorter than that of the ST time (56.92 ± 2.29 s), and the correct DT number (46.19 ± 0.69) was greater than that of the ST (44.15 ± 0.91) (26 cases analyzed), showing significant differences (*P* < 0.001 and 0.018, respectively).

### Hemodynamic changes

According to the results of the analysis of variance, compared with the baseline value in the standing position, the hemodynamics of PFC Δ[oxy-Hb] did not significantly change when walked on a walker at a normal pace, but during the cognitive tasks, both cognitive ST and DT significantly increased.

**Fig. 3:**
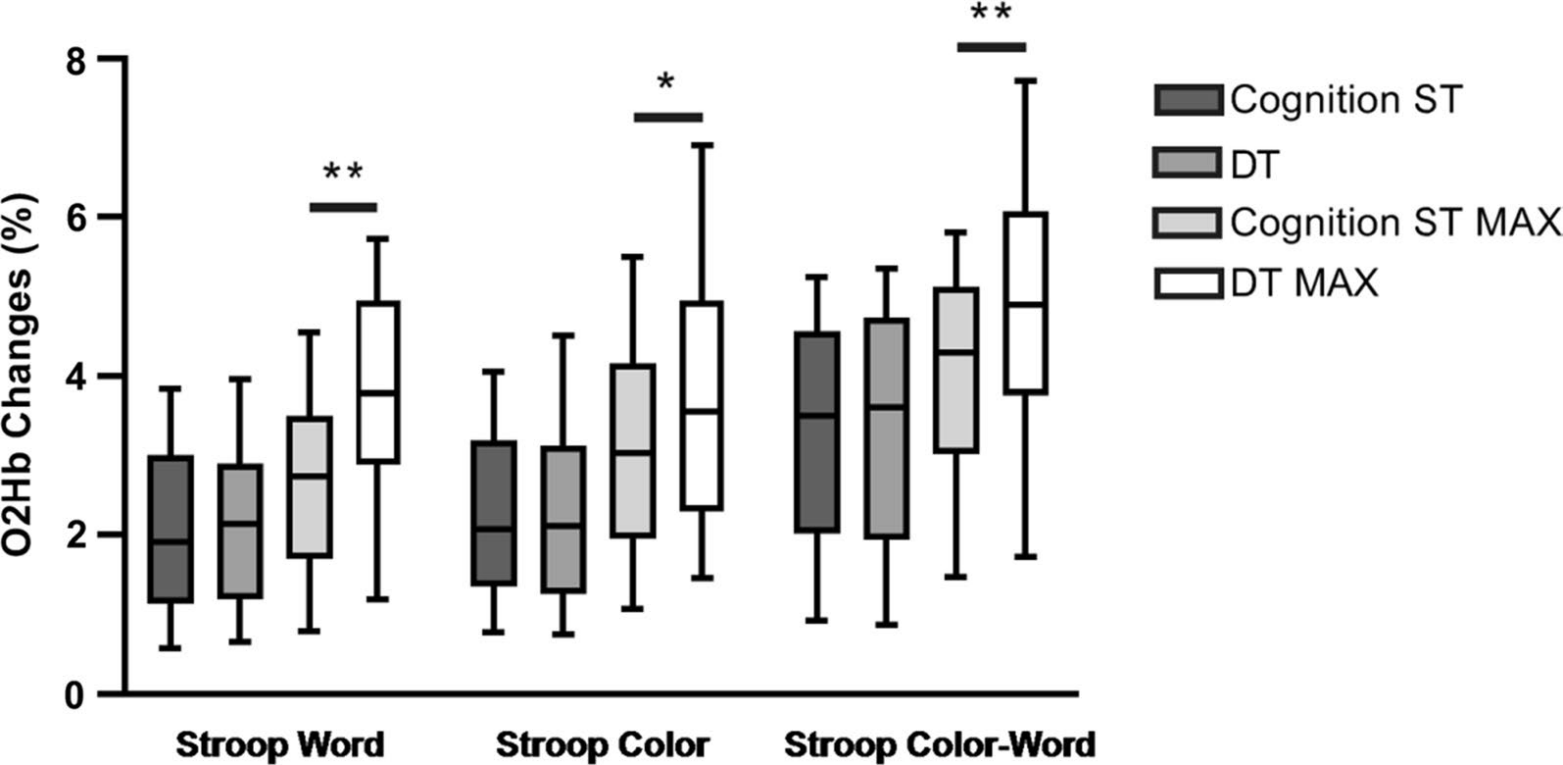
Changes of PFC brain oxygen parameters. MAX: the peak value of Δ[oxy-Hb]; *P < 0.05; **P < 0.01

**Fig. 4:**
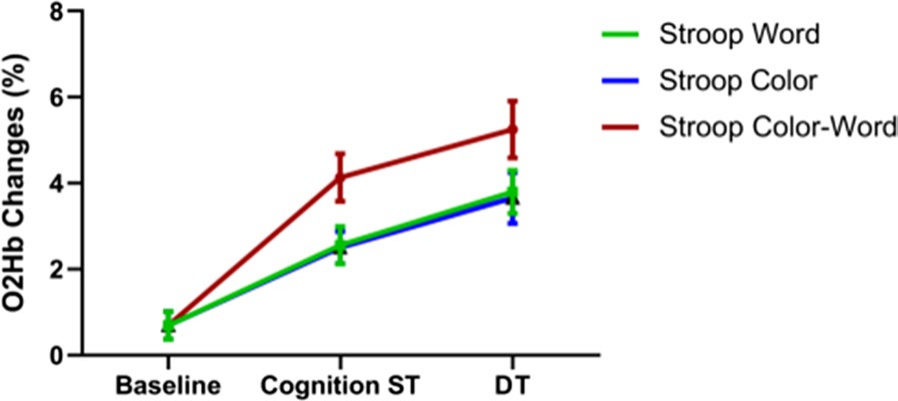
Changes of brain oxygen parameters during stroop test

There was no significant difference in the mean value of Δ[oxy-Hb] between the cognitive ST and DT (Stroop Word: 1.93 ± 0.39 and 2.25 ± 0.50, *P* > 0.05, Stroop Color: 2.12 ± 0.48 and 2.21 ± 0.52, *P* > 0.05, Stroop Color-Word: 3.41 ± 0.52 and 3.42 ± 0.61, *P* > 0.05, respectively), but the peak value of Δ[oxy-Hb], DT was significantly greater than that of the cognitive ST, which was present in three parts of the Stroop test (Stroop Word: 2.56 ± 0.43 and 3.80 ± 0.50, *P* < 0.01, Stroop Color: 2.50 ± 0.37 and 3.66 ± 0.59, *P* < 0.05, Stroop Color-Word: 4.13 ± 0.55 and 5.25 ± 0.66, *P* < 0.01, respectively) (Figs. 3, 4).

For the Δ[deoxy-Hb] and THI of the PFC, there was no significant change in each stage.

## Discussion

This study investigated the effects of walking on the execution abilities (Stroop test) of young participants. RPE and HR confirmed that it was always controlled at a low exercise intensity, involving the normal pace of daily walks. The main findings indicated that simple daily walking promoted performance of the Stroop Color-Word test, which was reflected in a shorter total reaction time and a higher correct number.

Physical exercise is one of the best non-drug methods to delay cognitive decline and can help improve executive functions [22, 23]. In this study, low intensity exercise had a positive effect on cognitive functions (i.e., executive functions), which was reflected in the participants’ faster response speeds and fewer errors. These results supported the findings of previous studies. Acute aerobic exercise can lead to positive physiological adaptations in the central nervous system and stimulate cognitive abilities after aerobic exercise, especially the ability to interfere with control, which is an important part of executive functions.

When analyzing the data of the cognitive test, we deleted the cases in which the Stroop test got full marks in the ST and DT test stages, which led to the fact that the number of analyzed cases for the Stroop test was not 30. The Stroop test is associated with educational level [24], and participants in this study are all university graduates or students with a high level of education, and may be too easy for them, so some cases get full marks. To avoid data bias caused by ceiling effects, we removed cases with full scores on both tests, resulting in a smaller data sample when analyzing Stroop word test and Stroop color test, and the meaningless results (*P* > 0.05) based on this may be unreliable. Subsequent experiments may increase the difficulty of cognitive tests or recruit participants of different educational levels. Subsequent experiments may increase the number of participants, with different levels of education, and increase the difficulty of cognitive tests.

A previous study reported that higher exercise intensity (vigorous intensity or high intensity interval training) could benefit more from acute aerobic exercise [25]. However, this study found that even low intensity exercise (45% HRmax) had beneficial cognitive effects. Previous studies generally believed that exercise would increase cerebral blood flow, and that cerebral blood flow was related to the degree of physical exertion [26–28], but there were also some contradictory results [29]. This is mainly due to the different physical exercise regimens used in different research protocols. At higher exercise intensity and physical exertion, hypercapnia due to hyperventilation, and sympathetically mediated protective mechanisms against excessive increases in cerebral blood flow, etc., can cause cerebral vasoconstriction at higher exercise intensity, so that the plateau period is even reduced [30]. Exercise-induced reductions in cerebral blood flow have been observed even at moderate exercise intensities [31]. The participants walked at a normal speed throughout the experiment, without causing significant changes in heart rate. By monitoring the cerebral oxygen parameters of the PFC, there was no significant change in the THI value, indicating that there was no significant change in the total hemoglobin concentration in the local brain tissue, reflecting that walking at a normal speed or Simultaneous Stroop testing did not cause a decrease in PFC tissue blood flow. Studies have shown that the RT of the Stroop test in young participants was related to HbO2 in the PFC, and that a faster RT was related to higher HbO2 availability [9]. We therefore suggest that the beneficial effect of walking on cognition may be due to higher peak cerebral oxygen, which has nothing to do with changes in systemic hemodynamics, because the THI value of the PFC during walking did not increase significantly compared with the resting state. The higher peak cerebral oxygen may therefore originate from the local self-regulation of cerebral blood vessels [32, 33].

Walking tasks occupy prefrontal lobe resources [34], and more complex walking tasks (such as obstacle walking) affect the activation of the prefrontal lobe, leading to poor performance in cognitive tasks [35]. To reduce the impact of the task difficulty of walking on the activation of the prefrontal lobe, the subjects completed the routine walking speed on a horizontal treadmill. Increased Oxy-Hb values have been shown to be associated with increased activation of responsive neurons [36]. Compared with the other tasks, the peak value of Δ[oxy-Hb] in the PFC of young adults was significantly increased during the dual task with Stroop Color-Word. Probably because walking or taking the Stroop color and Stroop Word tests simultaneously is a simple motor or cognitive task that does not require additional activation of the PFC. Actively increasing PFC activation may help prevent or delay age-related brain changes [37, 38], and regular dual-task training may delay age-related cognitive decline.

In addition to the contribution of higher brain oxygen levels to cognition, exercise may promote cognition in other ways. Stritt reported that exercise increased serotonin (5-HT) levels, even with low intensity exercise [39], and 5-HT played an important role in many cognitive processes. Tsai reported that acute exercise-induced neurocognitive changes were associated with real-time changes in circulating levels of neuroprotective growth factors, and acute exercise increased the amplitude of the event-related potential, P3 [40].

Exercise has beneficial effects on cognitive function [41]. The incidence of cognitive impairment is low in the younger population, but cognitive impairment increases with age [42, 43]. Previous research suggests that physical activity in youth may contribute to the development of healthy adult lifestyles, and physical activity appears to have long-term benefits on cognition, mental health, bone health, and sedentary behavior [43]. Our study found that low-intensity exercise such as normal pace walking has acute beneficial effects on cognitive function in young adults. Normal pace walking does not require special sports equipment or sports venues. It is a simple physical activity that can be carried out at any time. For people who lack physical exercise or cannot exercise vigorously due to obesity, disease, etc., walking can be used as one of the regular training modes.

## Limitations

In the present study, the treadmill speed of each participant was fixed when adjusted during the preparatory phase. Constant speed may affect changes in gait parameters. However, studies have shown that the motor systems of young participants had strong adaptabilities. When performing cognitive tasks at the same time, there was no significant change in gait parameters [44], which is consistent with our results. To reduce the interference of cognitive tasks, the experiments were conducted in a quiet room, which was not consistent with a daily walking environment. A complex environment affects cognition [45, 46], so future studies will include an environment resembling that in normal life.

The sample size of this study was small, and the population consisted mostly of healthy young participants. Studies have shown that exercise had lower cognitive gains for the elderly, and whether the conclusions of this study are applicable to the elderly remains to be further studied. Moreover, we have considered some deficiencies in the methodology. The NIRS system we used only allowed measurements in a limited area of the PFC, and current studies have shown that the functional activity of other areas of the brain changed during exercise [47]. Future research may use a multi-channel NIRS device to include a larger area. The NIRS equipment used in this study with a low sampling frequency, resulting in very limited data points on cerebral oxygen parameters, and equipment with a sampling frequency of 50 Hz or even higher can be used in the future. And this study conducted research from the perspective of cerebral hemodynamics, which can be further explored and analyzed from the perspective of neuroelectrophysiology and biomarkers.

## Conclusion

Normal-paced walking had beneficial effects on executive ability of young participants. By monitoring the hemodynamics of bilateral PFC during walking, this benefit may have been related to the peak cerebral oxygen of DT. However, the results of this study were limited, and it is still necessary to increase the types of participants and to improve monitoring techniques to improve the breadth of applications of the conclusions and mechanisms of the research.

## Data Availability

The datasets used and analyzed during the current study are available from the corresponding author upon reasonable request.
